# High prevalence of poor sleep quality and sleep deficit: A study in children, adolescents, and adult soccer players

**DOI:** 10.1371/journal.pone.0333774

**Published:** 2025-10-10

**Authors:** Lúcio A. Cunha, Elisa A. Marques, João Brito, Michele Lastella, Pedro Figueiredo

**Affiliations:** 1 Research Center in Sports Sciences, Health Sciences and Human Development, CIDESD, University of Maia, Maia, Portugal; 2 College of Sport Science, University of Kalba, Kalba, Sharjah, United Arab Emirates; 3 FPF Academy, Portuguese Football Federation, Cruz Quebrada, Portugal; 4 Appleton Institute, Central Queensland University, Adelaide, Australia; 5 Physical Education Department, College of Education, United Arab Emirates University, Al Ain, United Arab Emirates; Portugal Football School, Portuguese Football Federation, PORTUGAL

## Abstract

**Objective:**

This observational study aimed to provide insight into the sleep behaviors, chronotypes, and sleep needs of athletes by examining children, adolescents, and adult soccer players from different competitive levels.

**Methods:**

The study included 864 soccer players (n = 747 males) of various age groups [median age: 17 (interquartile range: 14–20)]. The participants completed an online questionnaire that included demographic questions, the Pittsburgh Sleep Quality Index (PSQI), the Epworth Sleepiness Scale (ESS), the Morningness-Eveningness Questionnaire (MEQ), and a question about their sleep needs.

**Results:**

Adult players had a higher prevalence of poor sleep quality, excessive daytime sleepiness, and sleep deficit than children and teenagers (p < 0.001). Lower sleep duration was associated with poorer sleep quality and excessive daytime sleepiness (r_s_ = −0.59 to −0.17, p < 0.01). Sleep needs were significantly higher than the habitual sleep duration in teenagers (42 min, p < 0.001) and adult players (41 min, p < 0.001). Players that had poor sleep quality (OR = 3.98, 95% CI: 2.78–5.77), excessive daytime sleepiness (OR = 3.72, 95% CI: 2.32–6.1), evening chronotype (OR = 2.54, 95% CI: 1.48–4.40), and later ending time of training (OR = 1.12, 95% CI: 1.07–1.19) could be at a higher risk of having sleep deficit.

**Conclusion:**

Nearly half of the adult players had poor sleep quality. Additionally, one-third of the children, and almost half of the teenagers and adult players, had a sleep deficit. Players with poor sleep quality, an evening chronotype, excessive daytime sleepiness, and a later training time might be at a higher risk of experiencing a sleep deficit.

## Introduction

The growing concern about athletes’ sleep has been reflected in the exponential increase in literature about sleep in sports [1]. Sleep is essential for human health due to its restorative qualities, and athletes recognize it as one of the most important recovery strategies [2]. Although data examining sleep in athletes is contentious, there is evidence indicating acute sleep restriction (less than 6 hours of sleep) is sufficient to impair athletes’ performance the following day [3]. Tasks requiring a skill component appear to be particularly sensitive to the effects of sleep loss (e.g., tennis serving) [3].

Currently, there is no specific recommendation on how much sleep athletes need to achieve optimal performance. Given the physiological and psychological stress imposed by sports environments, it may be reasonable that athletes’ sleep needs might differ from those of non-athletes, despite the lack of studies comparing sleep necessities between athletes and non-athletes [4]. At present, there is only one study that directly examined the amount of sleep athletes need, indicating that elite athletes perceived needing about 8.3 (0.9) hours of sleep to feel rested [5].

While the importance of sleep for performance and recovery is frequently emphasized by coaches and athletes [2,6], it has often been reported that athletes experience high levels of sleep disturbance (such as poor sleep quality, short sleep duration, inadequate sleep to meet their needs) [5,7–9]. For example, Halson et al. [7] showed that 52% of elite athletes had poor sleep quality. In another study, Merayo et al. [8] demonstrated that 70% of children and teenage soccer players sleep less than the minimum general recommendation. At the same time, Sargent et al. [5] showed that only 3% of adult elite athletes obtain enough sleep to satisfy their needs.

Although considerable research has been conducted on the sleep habits of soccer players, only small samples have been examined [10–15]. To date, only one large study investigated the sleep habits of young soccer players (n = 261) [8]. Still, this study investigated elite academy players, who may not be representative of all soccer players. Furthermore, it remains to be clarified whether certain variables (e.g., chronotype, training schedules, or competitive level) can influence the quality and duration of sleep that soccer players achieve.

While polysomnography is considered the gold standard for objectively examining sleep [16,17], it has some disadvantages, such as the cost of the equipment, the expertise required, and the unnatural sleep environment [16]. Given the disadvantages of polysomnography, some alternatives, such as validated questionnaires, are typically used to study sleep/wake behavior in athletes [16]. Some common questionaries used in sleep research are the Pittsburgh sleep quality index (PSQI), which assesses sleep quality [18], the Epworth sleepiness scale (ESS) used to evaluate daytime sleepiness [19] and the Morningness-eveningness questionnaire (MEQ), which can be used to identify the chronotype [20]. Despite some disadvantages (e.g., subjectivity or memory bias), questionnaires are cost and time-effective tools that make it more accessible to conduct observational studies with larger samples.

The aims of the present study were: 1) to describe the sleep quality, daytime sleepiness, chronotypes, and self-assessed sleep needs of soccer players; 2) to explore the relationship between sleep duration, sleep quality, and daytime sleepiness; and 3) examine the differences between players sleep needs and habitual sleep duration; 4) examine individual and contextual factors potentially related to sleep deficit.

## Materials and methods

### Participants and study design

A total of 864 soccer players volunteered to participate in this cross-sectional study. All players were required to participate in an official competition organized by the Portuguese Football Federation or regional football associations. Players’ competitive level was classified based on McKay et al. [18]. Players who competed at the national level were categorized as “national level”, and those who competed at the regional level were categorized as “developmental”. An online questionnaire (via Google Forms) was administered during the competitive phase of the seasons 2021/2022 and 2022/2023. Players were recruited from the Portuguese Football Federation database and through direct contact with the clubs. A link to access the questionnaire was sent directly to players (aged 16 years and above) via the Portuguese Football Federation newsletter on two occasions (May and October 2022) or shared with clubs between January 2022 and March 2023. Of the fourteen clubs contacted, nine agreed to share the questionnaire with players of different ages and teams.

### Ethical approval and informed consent

An electronic informed consent was obtained from players who were at least 16 years old. For players under 16 years old, written informed consent was obtained from parents and/or legal guardians. Players under 11 years old completed the questionnaire with help from a member of the coaching staff and/or the parents/legal guardians. The study was conducted in accordance with the Declaration of Helsinki and was approved by the ethics committee of the Portugal Football School, Portuguese Football Federation (nº 9/2021).

### Inclusivity in global research

Additional information regarding the ethical, cultural, and scientific considerations specific to inclusivity in global research is included in the Supporting Information (S1 Checklist).

### Questionnaire-derived sleep variables

The sleep habits were assessed using online Portuguese versions of the PSQI, the MEQ, and the ESS [19–21].

The PSQI is a validated 19-item self-rated questionnaire for evaluating subjective sleep quality in general and clinical populations over the previous month. It was developed to provide a reliable, valid, and standardized measure of sleep quality and to discriminate between “good” and “poor sleepers” [19]. It also assesses sleep duration derived from the reported time going to bed and waking up. Global scores range from 0 to 21, with higher scores indicating poorer overall sleep quality. A global PSQI score greater than 5 indicates a poor sleeper, while a score ≤5 indicates a good sleeper [22].

The MEQ assessed the chronotype, composed of 16 questions with scores ranging from 16 (peak of alertness in the afternoon/evening) to 86 (peak of alertness in the morning) points. A score between 43 and 53 indicates that the peak of alertness occurs at an intermediate time of day [20]. We selected the MEQ for this study because it is a well-validated and widely used instrument appropriate for heterogeneous samples. Unlike other chronotype instruments, the MEQ assesses diurnal preferences rather than actual sleep behavior, making it particularly suitable for players who frequently train or compete on weekends when sleep patterns are influenced more by external demands than internal rhythms.

The ESS measures the general level of daytime sleepiness or average sleep propensity [21]. The questionnaire is composed of 8 questions and yields a global score ranging from 0 to 24. Scores between 0 and 10 are considered normal, while scores between 11 and 24 indicate excessive sleepiness.

The sleep needs of the players were assessed with the question, “How many hours of sleep do you need to feel rested?”. A one-hour or more difference between the habitual sleep duration and sleep needs was considered to indicate a sleep deficit [5,23]. For healthy sleep, the American National Sleep Foundation recommends that school-aged children (6–13 years old) should sleep 9–11 hours per night, teenagers (14–17 years old) 8–10 hours, and adults (18–64 years old) 7–9 hours [24]. Concerning sleep quality, a sleep latency below ≤30 minutes and a sleep efficiency above ≥85% is recommended, regardless of age group [25].

### Other variables

We considered additional independent variables that could be associated with players’ sleep habits, including age, sex, competitive level, training schedules, and number of weekly training sessions. These variables were assessed through additional questions incorporated into an electronic questionnaire.

### Statistical analysis

All analyses were stratified by age groups (8–13 years, 14–17 years, ≥ 18 years), following the age categories defined by the National Sleep Foundation [24], and competitive levels (developmental and national). Between-group differences in continuous variables were analyzed using generalized linear models via the glm function from the *stats* package [26], selecting link functions and distribution families according to each outcome’s characteristics. Estimated marginal means and pairwise contrasts were obtained with *emmeans* package [27]. Model adequacy was assessed via the Akaike Information Criterion and residual diagnostics. For categorical outcomes, Pearson’s chi-square tests (or Fisher’s exact test when expected counts were <5) were used to test overall group differences in categorical outcomes. When the chi-square test was significant, cellwise standardized Pearson residuals were converted to two-sided p-values to identify which cells contributed most to the association [28]. A linear mixed model analysis was performed to examine differences between sleep needs and habitual sleep duration. Analysis was conducted using the lmer function from the *lme4* package [29]. Estimated marginal means and post hoc pairwise comparisons were obtained with the *emmeans* package [27]. Models were fitted by restricted maximum likelihood (REML), and appropriateness was verified by inspecting residual Q–Q plots. The Spearman correlation (r_s_) analysis was applied to determine the relationships between sleep duration, sleep quality, and sleepiness levels. Magnitude of the effects were interpreted as trivial (r ≤ 0.10), small (r = 0.10–0.3), moderate (r = 0.3–0.5), large (r = 0.5–0.7), very large (r = 0.7–0.9) and almost perfect (r ≥ 0.9) [30]. A multivariable logistic regression model was used to estimate the association of several potential predictors with sleep deficit. In the first multivariate model, the odds ratios (OR) and their 95% confidence limits (CI) for sleep deficit included the following independent variables: age, competitive level, end time of training, habitual bedtime, sleep quality, sleepiness level, and chronotype. In the second model, only variables significantly contributing to the multivariate model (retention threshold of P < 0.10) were included in the final analyses. Age and competitive levels were excluded from the final model because they were not significant predictors of sleep deficit (P ≥ 0.10) in the multivariate models. The effect was deemed non-significant when the 95% CI overlapped positive and negative values. Benjamini and Hochberg’s [31] procedure was applied for multiple testing corrections, and the FDR adjusted p-values were reported. FDR adjusted p-values lower than 0.05 were assumed to be statistically significant. All statistical analyses were performed in RStudio (version 2024.12.1 + 563, R Foundation for Statistical Computing, Vienna, Austria), and figures were created using GraphPad Prism 10 (GraphPad Software, San Diego, CA, USA).

## Results

### Participants characteristics

Of the 864 players included in this study, 747 (85%) were male and 117 (15%) were female, and the median age was 17 (interquartile range, 14–20 years). The age distribution of all study participants is illustrated in S1 Fig (Supplementary file). The majority (61%) were at the developmental level, and the training schedule was held in the afternoon or evening in all age groups. There were significant differences in the number of training sessions per week and training schedules across all age groups (p < 0.001). Table 1 shows the demographic details of the players by age group.

**Table 1:** Participants’ characteristics by age group.

	Age Groups, years			*p*-value
	8-13	14-17	≥18	
Competitive level, n (%)				
Developmental	169 (99%)^b^	184 (60%)	176 (46%)^b^	<0.001
National	3 (1%)^a b^	124 (40%)	208 (54%)^b^	
Sex, n (%)				
Male	172 (99%)^b^	270 (88%)	291 (76%)^b^	<0.001
Female	1 (1%)^b^	38 (12%)	93 (24%)^b^	
No. training sessions per week	3.03 (2.78–3.30)	3.58 (3.38–3.80)*	3.95 (3.76–4.14)* ^#^	<0.001
Training Schedules, h:min				
Start time	18:54 (18:24–19:24)	19:18 (18:54–19:42)	17:30 (17:12–17:48)* ^#^	<0.001
Finish time	20:12 (19:30–20:54)	20:54 (20:36–21:18)	19:06 (18:48–19:30)* ^#^	<0.001

Values of the continuous variables are marginal means and 95% confidence intervals. Values of categorical variables are n (%). ^a^ There is no national competition for this age group in Portugal. These players compete in a higher age group competition; ^b^ FDR-adjusted p < 0.05 from cellwise standardized residuals; * significantly different from 8-13y age group; # significantly different from 14-17y age group.

### Sleep behaviors, chronotypes, and needs

Players’ sleep behaviors (based on the PSQI and ESS), chronotypes, and sleep needs are summarized in Table 2. Significant differences between age groups were found in all outcomes (p < 0.001). When data was stratified by competitive levels, only the bedtime, sleep duration and the prevalence of excessive daytime sleepiness were different between groups (p < 0.001).

**Table 2:** Players’ sleep habits (quantity, quality, sleepiness), chronotype, and needs by age groups and competitive level.

	Age groups, years			*p*-value	Competitive Level		*p*-value
	8-13	14-17	≥18		Dev.	National	
Bed time (h:min)	22:15 (22:07–22:22)	23:10 (23:05–23:16)*	23:46 (23:41–23:52)* ^#^	<0.001	23:07 (23:01–23:12)	23:39 (23:22–23:35)	<0.001
Wake time (h:min)	7:39 (7:29–7:49)	7:28 (7:22–7:35)	8:02 (7:55–8:08)* ^#^	<0.001	7:46 (7:41–7:52)	7:44 (7:36–7:51)	0.607
Latency							
≤15 min	103 (59.9%)	206^a^ (66.9%)	197^a^ (51.3%)	<0.001	308 (58.25)	198 (59.1%)	0.647
16-30 min	57 (33.1%)	90 (29.2%)	141 (36.7%)		182 (34.4%)	106 (31.6%)	
31-60 min	11 (6.4%)	10^a^ (3.2%)	38^a^ (9.9%)		32 (6.0%)	27 (8.1%)	
>60 min	1 (0.6%)	2 (0.6%)	8 (2.1%)		7 (1.3%)	4 (1.2)	
Sleep duration (h)	8.9 (8.7–9.1)	7.8 (7.7–7.9)*	7.4 (7.3–7.5)* ^#^	<0.001	8.0 (7.9–8.1)	7.6 (7.5–7.7)	<0.001
Meeting sleep duration guidelines	103 (60%)^a^	198 (64%)^a^	311 (81%)^a^	<0.001	359 (68%)	253 (76%)	<0.05
Poor SQ	16 (9%)^a^	57 (18%)^a^	165 (43%)^a^	<0.001	128 (24%)	110 (33%)	0.221
ED sleepiness	5 (3%)^a^	43 (14%)	82 (21%)^a^	<0.001	60 (11%)	70 (21%)	<0.001
Chronotype							
Morning	26 (33%)^a^	48 (16%)	73 (19%)	<0.001	75 (17%)	72 (21%)	0.248
Intermediate	46 (58%)	194 (64%)	221 (58%)		270 (63%)	191 (58%)	
Evening	7 (9%)^a^	62 (20%)	90 (23%)		87 (20%)	72 (21%)	
Sleep needed (h)	8.9 (8.7–9.1)	8.5 (8.4–8.6)*	8.1 (8.0–8.2)* ^#^	<0.001	8.4 (8.3–8.5)	8.3 (8.2–8.4)	0.159
Sleep deficit	23 (29%)^a^	136 (45%)	177 (47%)	0.020	184 (43%)	152 (46%)	0.600

Abbreviations: Dev = Developmental, SQ =Sleep quality, ED= Excessive daytime

Values of the continuous variables are marginal means (95% confidence intervals). Values of categorical variables are n (%). ^a^FDR-adjusted p < 0.05 from cellwise standardized residuals. * Significantly different from 8-13y age group; # significantly different from 14-17y age group.

The percentage of missing values for sleep needs was 54.7%, 2.9%, and 1.8% for the age groups 8-13y, 14-17y, and ≥18y, respectively; and on the MEQ was 54.1% in the age group 8-13y, and 1.3% in the age group 14-17y.

### Correlations between sleep duration, sleep quality, and sleepiness

The correlation for the entire sample revealed that having worse sleep quality was moderately correlated with higher levels of sleepiness (r_s_ = 0.40, *p *< 0.001) and largely correlated with shorter sleep duration (r_s_ = −0.54, *p *< 0.001). Additionally, the results showed that less sleep duration was moderately correlated with higher levels of sleepiness (r_s_ = −0.34, *p *< 0.001). The correlations between sleep duration, sleep quality and sleepiness stratified by age group and competitive level showed similar results (Table 3).

**Table 3:** Correlations between sleep duration, sleep quality, and sleepiness stratified by age group and competitive level.

	Sleep quality & Sleepiness	Sleep quality & sleep duration	Sleepiness & sleep duration
**Age Groups**			
*8-13 y* *(n = 172)*	*r*_*s*_* *= 0.20, *p = *0.01, small	*r*_*s*_* *= −0.38, *p *< 0.001, moderate	*r*_*s*_* *= −0.19, *p = *0.01, small
*14-17 y* *(n = 308)*	*r*_*s*_* *= 0.36, *p *< 0.001, moderate	*r*_*s*_* *= −0.43, *p *< 0.001, moderate	*r*_*s*_* *= −0.17, *p = *0.002, small
*≥18 y* *(n = 384)*	*r *= 0.31, *p *< 0.001, moderate	*r*_*s*_* *= −0.57, *p *< 0.001, large	*r*_*s*_* *= −0.21, *p *< 0.001, small
**Competitive Level**			
*Developmental (n = 529)*	*r*_*s*_* *= 0.39, *P *< 0.001, moderate	*r*_*s*_* *= −0.50, *P *< 0.001, large	*r*_*s*_* *= −0.34, *P *< 0.001, moderate
*National* *(n = 335)*	*r*_*s*_* *= 0.36, *P *< 0.001, moderate	*r*_*s*_* *= −0.59, *P *< 0.001, large	*r*_*s*_* *= −0.26, *P *< 0.001, small

### Differences between sleep need and habitual sleep duration

**Fig 1:**
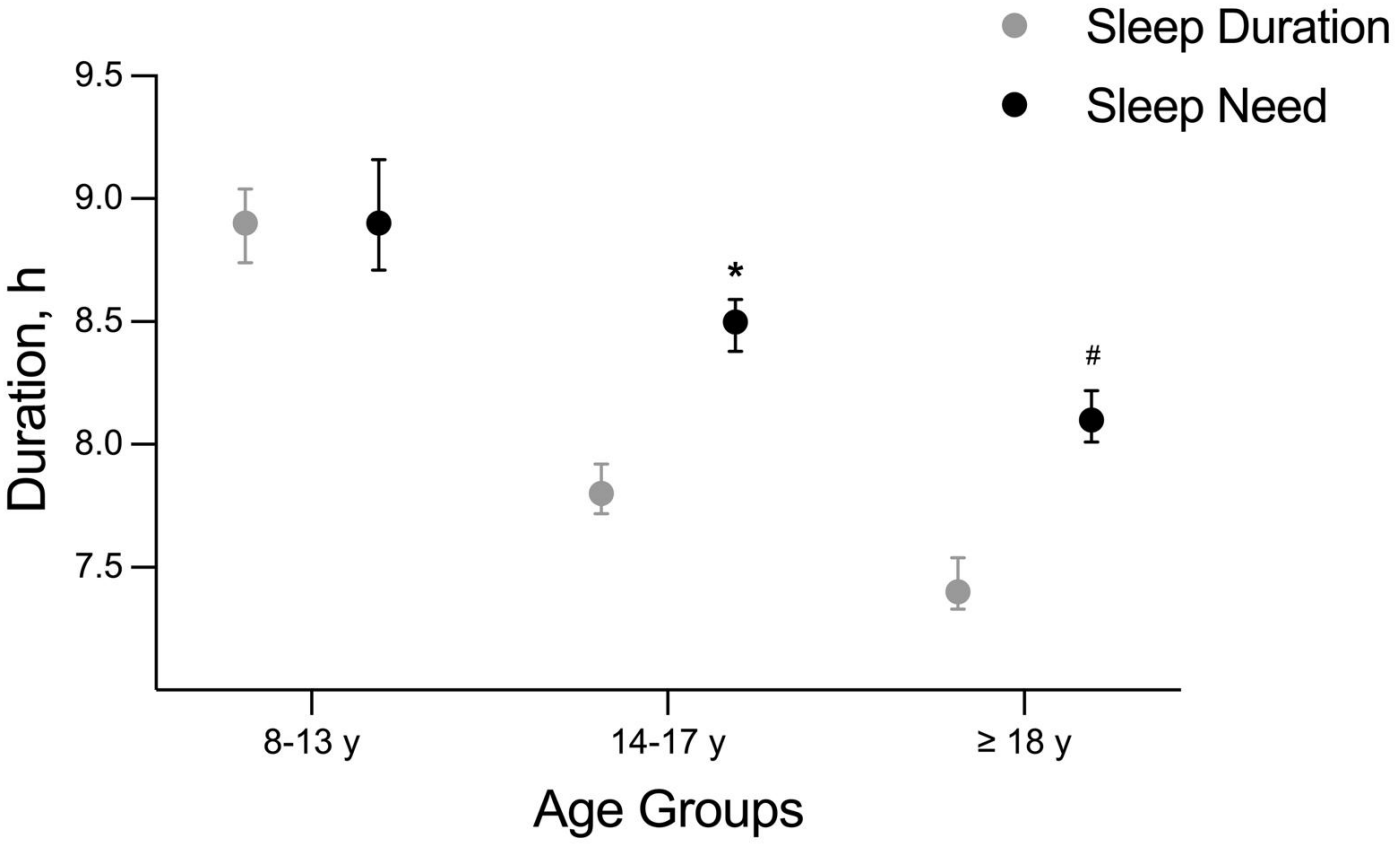
Self-assessed sleep needs compared with habitual sleep duration in players by age groups. Data are marginal means and 95% confidence intervals. * Significantly different from sleep duration within the 14–17 y age group (p < 0.001), ^#^ Significantly different from sleep duration within the ≥ 18y age group (p < 0.001).

The habitual sleep duration was significantly lower than the self-perceived sleep needs in the age groups 14-17y (−42 min, CI: −48–-32; *p *< 0.001), ≥ 18y (−41 min, CI: −48–-33; *p *< 0.001) and total sample (−31 min, CI: −37–-26; *p *< 0.001). Fig 1 illustrates the comparisons in the three age groups [8–13 y (n = 78), 14–17 y (n = 299), ≥ 18 y (n = 377)].

### Variables associated with sleep deficit

**Fig 2:**
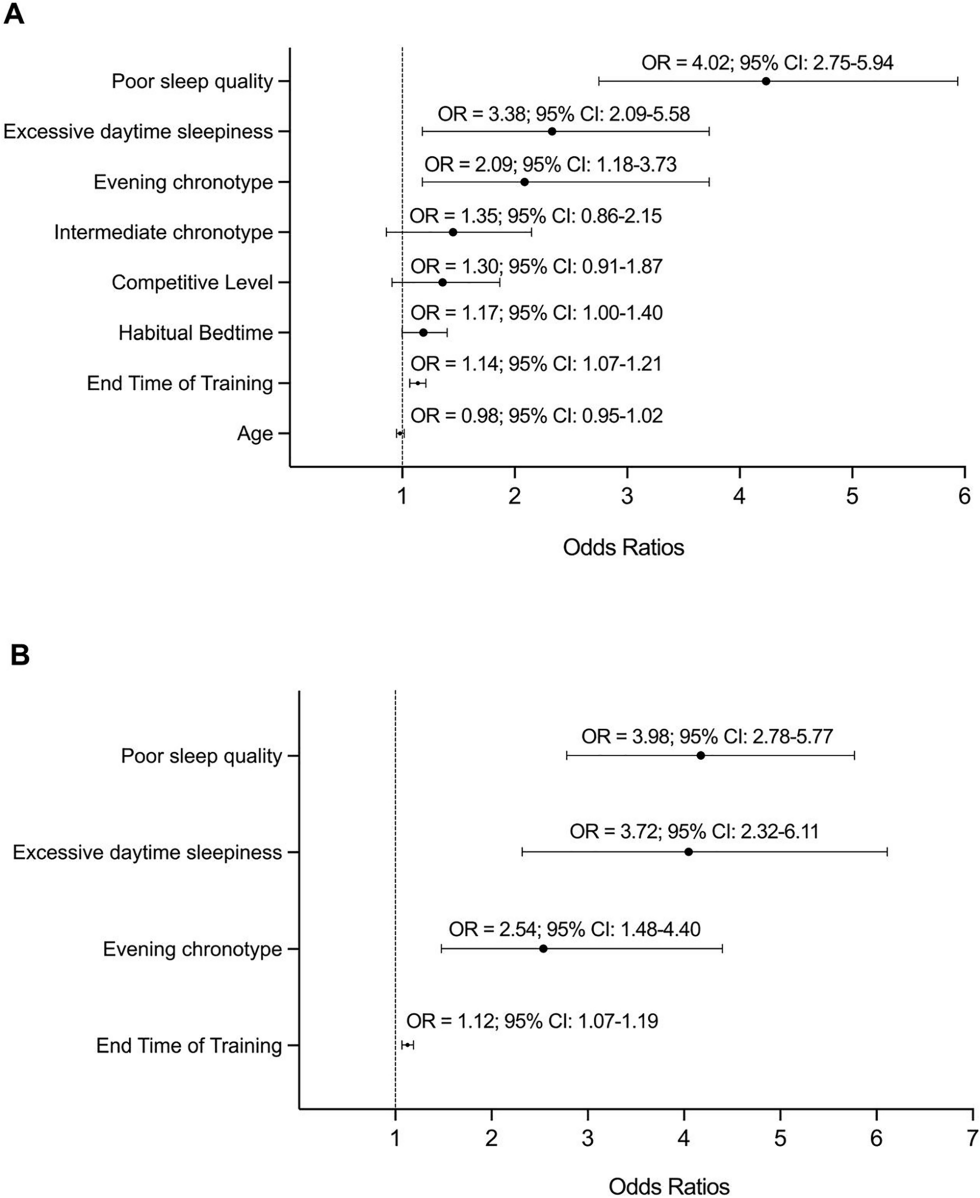
Associations between potential predictors and sleep deficit estimated by logistic regression models. A, Odds ratios and 95% confidence limits of model 1 (all potential predictors); B, Odds ratios and 95% confidence limits of model 2 (refined model retaining only significant predictors, p < 0.10). The dashed line represents the odds ratio = 1.

Fig 2 summarizes the results of the two models in the logistic regression analyses for sleep deficit. Model 1 included sleep quality, sleepiness level, chronotype, end time of training, and habitual bedtime and was adjusted to age and competition level. Only sleep quality, sleepiness level, chronotype, and end time of training were significant (*p* < 0.05). Model 2, which included sleep quality, sleepiness level, chronotype, and end time of training, was significantly better than baseline in explaining sleep deficits [X^2^(5)= 168.53; *p *< 0.001]. Among the associated factors with sleep deficit, poor sleep quality showed the highest association (OR = 3.98, 95% CI: 2.78–5.77), followed by excessive daytime sleepiness (OR = 3.72, 95% CI: 2.32–6.1), evening chronotype (OR = 2.54, 95% CI: 1.48–4.40), and end time of training (OR = 1.12, 95% CI: 1.07–1.19).

## Discussion

This large observational study of soccer players revealed key findings regarding age-related sleep patterns and associated factors. Adult soccer players (≥18 y) demonstrated substantially higher prevalence of poor sleep quality (43%) compared with younger players (8–17 years), along with higher rates of excessive sleepiness (21%) and sleep deficit (47%) relative to the youngest cohort (age 8–13 years), suggesting a progressive decline in sleep parameters with advancing age. Most players exhibited intermediate chronotype. Correlation analyses revealed interconnected relationships where poor sleep quality was associated with increased daytime sleepiness and reduced sleep duration, while shorter sleep duration correlated with higher daytime sleepiness levels. Notably, significant discrepancies between sleep needs and actual (habitual) sleep duration were evident in teenage and adult players, with those exhibiting poor sleep quality, excessive daytime sleepiness, evening chronotype, and later training schedules demonstrating significantly higher risk of sleep deficit, suggesting multiple contributing factors to inadequate sleep in this athletic population.

Consistent with previous research [7,32], the current investigation revealed that 43% of adult soccer players have poor sleep quality. Mah et al. [32], in a study with collegiate student-athletes, showed that 42% of the athletes were identified as poor sleepers (PSQI>5), similar to Halson et al. [7] in elite Australian athletes (52%). Regarding young soccer players, Merayo et al. [8] found that, on average, the quality of sleep was poor (n = 261, average age: 13.04 ± 3.16) and worse in young adults (16-25y) and teenage players (12-15y) compared with children players (7-11y). Our results confirm that teenagers and adult players have poorer sleep quality than children. It is important to recognize that the study from Merayo et al. [8] used a different questionnaire to assess sleep quality (the sleep disturbance scale in children) [33]. While adult players had a higher proportion of poor sleep quality compared with youth players, this finding may be related to the fact that the adult players in this sample were not professional. The observed increase in training frequency with age may explain the high prevalence of poor sleep quality among adult players. Indeed, our older athletes (≥18 years) reported a higher prevalence of poor sleep quality than the prevalence reported by Portuguese high school students aged 16–23 years (36.6%) [34]. Although our study lacked a non-athlete control group, previous research suggests that elite athletes tend to report poorer subjective sleep quality than age-matched controls, despite having comparable total sleep duration [35]. These findings suggest that, in addition to age, sport-specific factors such as circadian misalignment and training demands may adversely affect athletes’ sleep quality. Our dataset included training frequency information across age groups; however, these data were insufficient for a comprehensive analysis of the training load-sleep relationship. Future investigations should examine this association using detailed training metrics (frequency, duration, and intensity). In addition, the consumption of stimulants such as coffee, tobacco, and alcohol was not assessed in this study; it is well known that their use tends to increase with age. This could be an additional factor contributing to the higher prevalence of poor sleep quality observed in adults, given the established negative impact of these substances on sleep parameters [36–38]. Another possible contributing factor is social jetlag, which refers to the misalignment between an individual’s biological clock and social obligations, often reflected in differing sleep schedules between workdays and free days. Non-professional players, who typically train late in the day, may be more susceptible to social jetlag, which has been linked to poorer sleep quality and overall health outcomes [39]. This high percentage of poor sleep quality should serve as a warning for players and clubs to pay closer attention to their sleep habits, as it is widely recognized that sleep is essential for recovery and performance [6,40].

Regarding chronotypes, the results showed that most players in our sample have an intermediate chronotype, and children have a higher proportion of morning types compared with teenagers and adult players. The findings are consistent with previous studies. Lastella et al. [41] demonstrated that 55% of female professional football players have an intermediate chronotype, and in another study among elite athletes from several sports, the prevalence of intermediate chronotype was 68% [42]. An athlete’s chronotype may influence the time of day of maximal performance, reflecting the variations in psychophysiological determinants of performance [41]. The misalignment of chronotype with training and competition times could result in poorer performance, higher perceived exertion, and increased fatigue during training sessions [41,43]. Roveda et al. [44] studied the effect of chronotype misalignment on performance in adolescent football players. They found that morning types performed better in the morning compared with the evening session, and the evening types performed better in the evening than in the morning session. In contrast, no differences were observed in the intermediate types. Despite this, the influence of chronotype misalignment on long-term training adaptations remains to be elucidated.

The present study revealed that 130 players (15%) experienced excessive daytime sleepiness, with the majority (63%) being adults. This prevalence was substantially lower than rates reported in other studies with athletes [45,46] and Portuguese adolescents [47] populations. Among athletic populations, Pereira et al. [45] reported 44.9% prevalence in individual and team sports athletes, while Swinbourne et al. [46] found 28% prevalence in highly trained rugby, cricket, and rugby sevens players. When compared to Portuguese non-athletic populations, our findings also showed lower rates. A large study of 6919 Portuguese high school students (aged 12–18 years) reported 33% experiencing excessive daytime sleepiness [47], more than double our observed prevalence. Similarly, in a younger Portuguese sample of 7^th^ and 8^th^ grade students (mean age 13 ± 0.9 years), 11% reported sleepiness using the Paediatric Daytime Sleepiness Scale [48], compared to only 3% in our younger athletes (8–13 years). These comparative findings suggest that excessive daytime sleepiness follows age-related trajectories and may not be exclusive to athletes, potentially reflecting broader sleep disturbances among Portuguese youth.

Our results showed an association between higher levels of daytime sleepiness, poorer sleep quality, and shorter sleep duration. This was supported by Merayo et al. [8], who also found an association between poor sleep quality and less sleep duration, despite measuring sleep duration with sleep diaries. Daytime sleepiness is a state that is theoretically explained by insufficient sleep or inadequate sleep quality. Ritland et al. [49] and Mah et al. [50] showed that extending sleep reduced daytime sleepiness. Players with excessive daytime sleepiness may benefit from extending sleep duration through nighttime sleep or naps.

Approximately one-third of the youngest cohort (8–13 years) and almost half of both teenagers (14–17 years) and adult players experienced a sleep deficit. Interestingly, when assessed against general sleep duration guidelines, 81% of adult players met the recommended amounts, despite their high sleep deficit, compared to only 64% and 60% of the younger cohorts, respectively. However, these guidelines were not specifically developed for athletes and may underestimate their self-perceived sleep needs. Compared to Portuguese non-athletic adolescents, the findings are somewhat inconsistent, with compliance rates for general sleep duration guidelines varying considerably from 55% [51] to 73% [52].

Analyzing the players with sleep deficit, the results revealed an average difference of 1.32 ± 0.55 hours between sleep needs and sleep duration. In a study examining elite adult athletes from multiple sports, the prevalence of sleep deficit was even higher (71%) compared to the 46% we observed in the present study. While there has been increasing attention to the impact of sleep deficit on physical performance, experimental studies have focused on more “extreme” sleep-restriction protocols (i.e., ≤ 6 h sleep in any 24-hour period) [3]. Future studies should investigate the effect of shorter sleep restriction (e.g., 1-2h) on physical and cognitive performance.

When exploring the potential factors associated with sleep deficit, data showed that having poor sleep quality (OR = 3.98; 95% CI: 2.78–5.77), excessive daytime sleepiness (OR = 3.72; 95% CI: 2.32–6.1), evening chronotype (OR = 2.54; 95% CI: 1.48–4.40) and later training end times (OR = 1.12; 95% CI: 1.07–1.19) were positively associated. Since chronotype is mainly influenced by non-modifiable factors (e.g., age, sex or photoperiod at birth) [53] and training schedules always depend on the club logistics, players should consider adopting strategies like napping, sleep extension, sleep hygiene, or mindfulness, which could result in better sleep quality and/or duration [54].

Although we believe that objective tools should always be the first choice for measuring sleep, validated questionnaires are a commonly used method in large observational studies, particularly when resources are limited (e.g., budget or time) or data are collected in real-world settings (such as athletes during competitive periods). In the practical context of sport, despite the limitations, questionnaires like PSQI, ESS, and MEQ, or sleep diaries, could be a reasonable alternative to evaluate the sleep behaviors of the athletes when there is no access to objective tools (e.g., actigraphy) [16]. These tools are simple to apply in all contexts, cost- and time-effective, available in several languages, and can identify athletes with sleep disturbances who may benefit from an intervention or should be referred, ideally, to a sleep specialist.

This study has several limitations that warrant consideration. First, we relied exclusively on self-reported measures for sleep outcomes and assessment of the circadian rhythm. While these instruments are validated, subjective measures may diverge from objective assessments, which could potentially affect the accuracy of the results. Second, missing MEQ and sleep needs data for participants aged 8–13 years limits our ability to draw definitive conclusions about circadian preferences and sleep deficits in this age group. Finally, the substantial sex imbalance (117 females vs. 747 males) precluded meaningful sex-based comparisons, as further stratification would have created subgroups too small for reliable statistical analysis. Nevertheless, the present study included a large sample size with a wide age range and different competitive levels, which can increase the generalizability of the findings. Additionally, the use of questionnaires that assessed sleep quality, daytime sleepiness, chronotypes, and sleep needs provided a more detailed description of sleep characteristics in soccer players.

## Conclusion

The sleep behavior of adult soccer players was poorer than that of younger players. Adult players had a higher prevalence of poor sleep quality, excessive daytime sleepiness, and sleep deficit (i.e., ≥ 1h difference between self-reported habitual sleep duration and sleep need). Sleep deficits were more prevalent in teenage and adult players than in children. In all age groups, most players had an intermediate chronotype. Lower sleep quality and reduced sleep duration were largely correlated, while lower sleep quality, higher daytime sleepiness, and reduced sleep duration were moderately correlated. Poor sleep quality, excessive daytime sleepiness, evening chronotype, and a later training ending time were all associated with a sleep deficit. Based on our findings, strategies designed to improve sleep duration seem to be a key aspect of sleep behavior interventions or counselling for soccer players, particularly in adults. These strategies should benefit from focusing on improving sleep quality and lowering daytime sleepiness.

## Supporting information

S1 Fig Age distribution of study participants. (TIFF)

S1 Checklist Inclusivity in global research. (DOCX)

S1 File Data. (CSV)
