# Supplementary figures and images for: High prevalence of poor sleep quality and sleep deficit: A study in children, adolescents, and adult soccer players
Source: PLoS One. 2025 Oct 10;20(10):e0333774. doi: 10.1371/journal.pone.0333774 (PMC12513668; doi:10.1371/journal.pone.0333774)

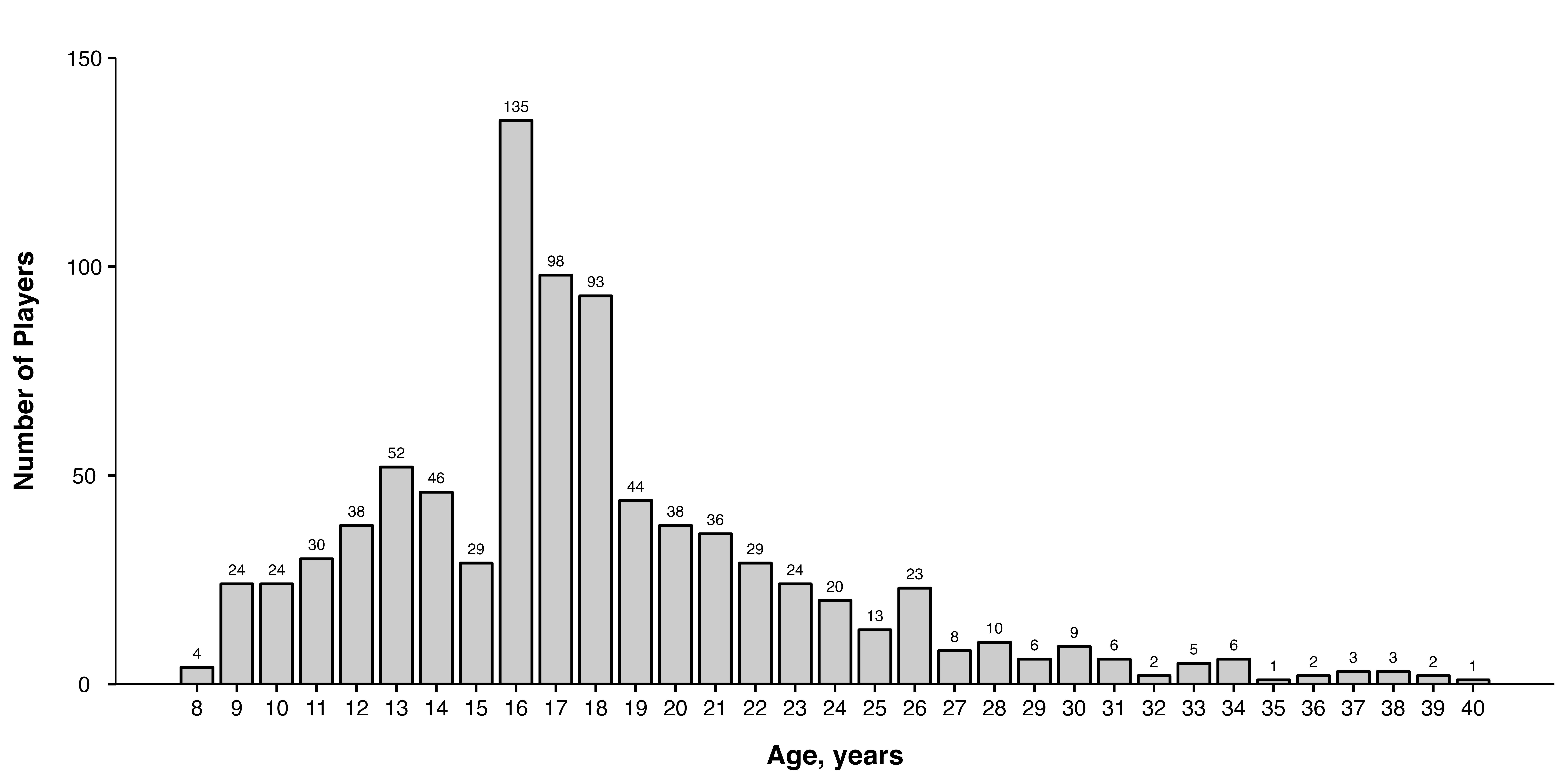

Supplement: S1 Fig — (TIFF) [file pone.0333774.s001.tiff]
